# In Silico Prediction of the Dissociation Rate Constants of Small Chemical Ligands by 3D-Grid-Based VolSurf Method

**DOI:** 10.3390/ijms21072456

**Published:** 2020-04-02

**Authors:** Shuheng Huang, Linxin Chen, Hu Mei, Duo Zhang, Tingting Shi, Zuyin Kuang, Yu Heng, Lei Xu, Xianchao Pan

**Affiliations:** 1 Key Laboratory of Biorheological Science and Technology (Ministry of Education), Chongqing University, Chongqing 400044, China; shhuang@cqu.edu.cn (S.H.); chenlinxin@cqu.edu.cn (L.C.); 2 College of Bioengineering, Chongqing University, Chongqing 400044, China; 20161902086@cqu.edu.cn (D.Z.); shitingting@cqu.edu.cn (T.S.); 201819021101@cqu.edu.cn (Z.K.); 201819021102@cqu.edu.cn (Y.H.); 201919021051@cqu.edu.cn (L.X.); 3 Department of Medicinal Chemistry, College of Pharmacy, Southwest Medical University, Luzhou 646000, China

**Keywords:** VolSurf, dissociation rate constant, Partial Least Squares, prediction

## Abstract

Accumulated evidence suggests that binding kinetic properties—especially dissociation rate constant or drug-target residence time—are crucial factors affecting drug potency. However, quantitative prediction of kinetic properties has always been a challenging task in drug discovery. In this study, the VolSurf method was successfully applied to quantitatively predict the *k_off_* values of the small ligands of heat shock protein 90α (HSP90α), adenosine receptor (AR) and p38 mitogen-activated protein kinase (p38 MAPK). The results showed that few VolSurf descriptors can efficiently capture the key ligand surface properties related to dissociation rate; the resulting models demonstrated to be extremely simple, robust and predictive in comparison with available prediction methods. Therefore, it can be concluded that the VolSurf-based prediction method can be widely applied in the ligand-receptor binding kinetics and de novo drug design researches.

## 1. Introduction

Although thermodynamic properties (e.g., IC_50_, EC_50_ and equilibrium dissociation constant (k_d_)) have been regarded as key indicators of drug potency, mounting evidence suggests that thermodynamic properties may not be the only measures of drug potency. Recently, it has become increasingly apparent that kinetic properties—especially dissociation rate constant (k_off_) or drug-target residence time (τ)—are more important for drug potency and are gradually being used in real-world lead optimization and drug design [1,2,3,4,5]. At present, kinetic properties are mainly determined by laboratory techniques, such as capillary electrophoresis, [6] affinity chromatography [7] and surface plasmon resonance methods [8,9], etc. However, there are still many technical difficulties need to be addressed, e.g., more time consumed, high cost and large measurement errors, which limit drug R&D to a large degree.

With the development of computational chemistry, molecular simulation techniques have been successfully applied to predict the binding kinetic properties of small molecules. One of the most common methods is molecular dynamics. By using τ-random acceleration molecular dynamics (τRAMD), Kokh et al. [10] proposed a protocol to predict the residence time of 70 inhibitors of human heat shock protein 90α (HSP90α). A strong correlation (R^2^ = 0.66) was observed between the predicted and measured residence time in 59 samples after removing 11 samples. After further removing four outliers, the *R*^2^ value of the linear regression of the remaining 55 compounds was 0.86 with a mean absolute percentage error (MAPE) of 0.36. In additions, the prediction method was further tested by 94 HSP90 inhibitors. The results showed that the predicted R^2^ of the 80 inhibitors was 0.75 with MAPE of 0.39 [11].

To investigate the quantitative structure-kinetics relationships (QSKRs) of 66 HSP90 inhibitors and 33 HIV-1 protease inhibitors, Ganotra et al. [12] proposed the “COMBINE” strategy that established PLS models by using 30 Lennard-Jones (LJ) and 12 coulombic residue-ligand interaction energies derived from the energy-minimized structures of drug-protein complexes. The results showed that *R*^2^, *Q*^2^ and *R^*2*^_val_* are 0.80, 0.69 and 0.86 for HSP90 inhibitors. By integrating coarse-grained normal mode analysis with multi-target machine learning, Chiu et al. [13] proved that the residue normal mode directionality displacement of receptor-ligand interactions can not only recapitulate the results from all-atom molecular dynamics simulations but also predict protein ligand binding/unbinding kinetics accurately.

Huang et al. [14] extracted ligand–receptor interaction energy fingerprints from the steered MD trajectories of 37 HIV-1 protease inhibitors, which were further used for estimating the ligand dissociation rate constants by partial least squares (PLS) regression successfully. By employing position-restrained molecular dynamics simulations, Zhang et al. [15] decomposed the protein-ligand interaction fingerprints alone the ligand-unbinding pathway and constructed PLS models to predict *k_off_* value of 20 p38 mitogen-activated protein kinase (p38 MAPK) Type II inhibitors. The result showed that the *R*^2^, *Q*^2^ and *R^*2*^_val_* of the optimal model with three descriptors are 0.72, 0.66 and 0.563, respectively.

Although MD simulations can provide a feasible way for predicting the receptor-ligand binding kinetics, its practical effectiveness is limited by the substantial computational resources required, underdeveloped MD force fields and relatively lower prediction accuracies. Thus, traditional ligand-based prediction method is still a first choice for predicting ligand binding kinetics, especially for lead compound optimization and virtual screening researches.

Recently, Qu et al. [16] employed a 3D grid-based VolSurf method to predict association rate constant (k_on_), dissociation rate constant and equilibrium dissociation constant (*k_d_*) values of 37 HIV-1 protease inhibitors with satisfactory results. The results showed that the kinetic properties of 37 HIV-1 protease inhibitors are closely related to the nine VolSurf descriptors derived from water (OH2) and hydrophobic (DRY) probes. In this study, we further examine the applicability and reliability of the VolSurf method by predicting the ligand dissociation rate constants of adenosine receptor (AR), HSP90 and p38 MAPK. The results showed that all of the three biologic systems achieved satisfied prediction performances. In general, the VolSurf method is easily implemented and can offer a practical and promising way for predicting ligand kinetic properties.

## 2. Results and Discussions

### 2.1. Heat Shock Protein 90α

HSP90 is a highly conserved molecular chaperone, which is involved in many different cellular pathways (e.g., the signal transductions of hormone and growth factor receptors) and maintains proteostasis involved in signal transduction, cell cycle control or transcriptional regulation [17,18]. Accumulated evidences suggested that Hsp90 is highly expressed in most of tumor cells in a high-affinity conformation [19]. Thus, HSP90 have been suggested as important therapeutic targets of cancer. In this study, 52 inhibitors of HSP90 with different molecular skeletons were derived from Schuetz’s research (Table S2) [20]. Herein, the 52 inhibitors were randomly divided into 35 training samples and 17 validation samples.

Based on the variable subsets derived from stepwise multiple regression (SMR), PLS modeling was performed. From Table 1, it can be observed that all of the nine PLS models achieved good prediction performances. In the consideration of model complexity and interpretability, the PLS model with only two descriptors (V-OH2 and D8-DRY) was chosen as the optimal PLS model, of which the *R*^2^, *Q*^2^ and *R^*2*^_val_* are 0.726, 0.688 and 0.718, respectively. The optimal PLS model suggests that the dissociation rate of HSP90 inhibitors are closely related to the molecular volume and hydrophobic properties.

**Figure 1:**
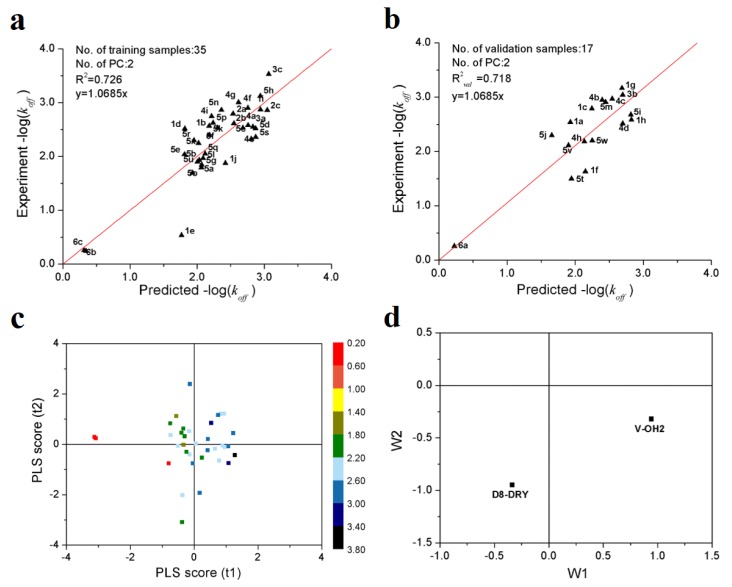
PLS modeling results of the dissociation rate constants of 52 Hsp90 inhibitors. (**a**) scatter plot of experimental vs. predicted −log(*k_off_*) of 35 training samples; (**b**) scatter plot of experimental vs. predicted −log(*k_off_*) of 17 validation samples; (**c**) first two principle component scores. The color legend represents the range of −log(*k_off_*) values; (**d**) loading plot of the first two principal components.

Figure 1a,b show the predicted vs. observed−log(*k_off_*) values of 35 training and 17 validation samples of Hsp90 inhibitors. It can be seen all the samples are distributed along the regression lines through the origin very well. It should be noted that, although the −log(*k_off_*) values differ by three orders of magnitude, all of the validation samples are predicted accurately. Figure 1c shows the first two principal component scores of the 35 training samples. It can be observed that, in first two principle component spaces, the experimental −log(*k_off_*) values increased gradually along the direction from the 3rd to 1st quadrants. From the loading scatter plot of the optimal PLS model (Figure 1d), it can be seen that, in the first principal component, V-OH2 variable makes positive contributions to −log(*k_off_*), while D8-DRY negative contributions.

**Figure 2:**
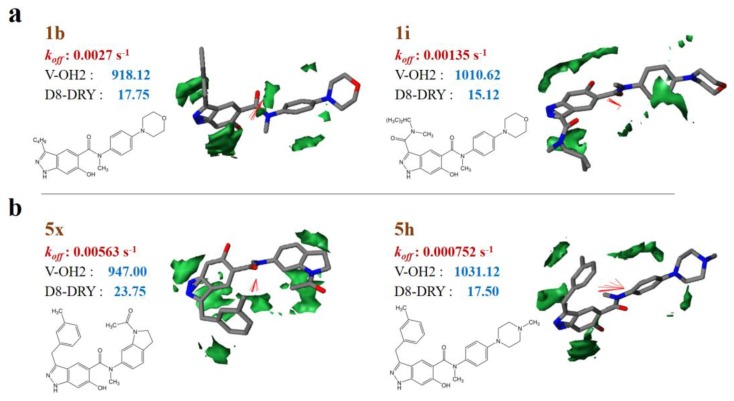
VolSurf properties of representative samples with different molecular skeletons. (**a**) 1b and 1i; (**b**) 5× and 5h. The hydrophobic regions at −1.6 kcal/mol energy level; red vectors represent the integy moments joining the center of mass of the molecule to the barycenter of the hydrophobic regions.

Taken sample 1i (1b) and 5h (5×) for example, each pair of samples are similar in structures, but with different dissociation rate constants. By comparison, 1i and 5h have lower dissociation rate constants than 1b and 5×, respectively (Table S2). It can be observed that the molecular volume of 1i and 5h are higher than that of 1b and 5× respectively, while the hydrophobic regions (−1.6 kcal/mol level) of 1i and 5h are relatively lower (Figure 2). That is to say, relatively larger molecular volume contributes to lower dissociation rate or longer residence time, while larger hydrophobic regions may benefit from higher dissociation rate or shorter residence time. It should be noticed that the strong correlation between the *k_off_* values and the molecular sizes of HSP90 inhibitors has been detailed in earlier research [21].

**Figure 3:**
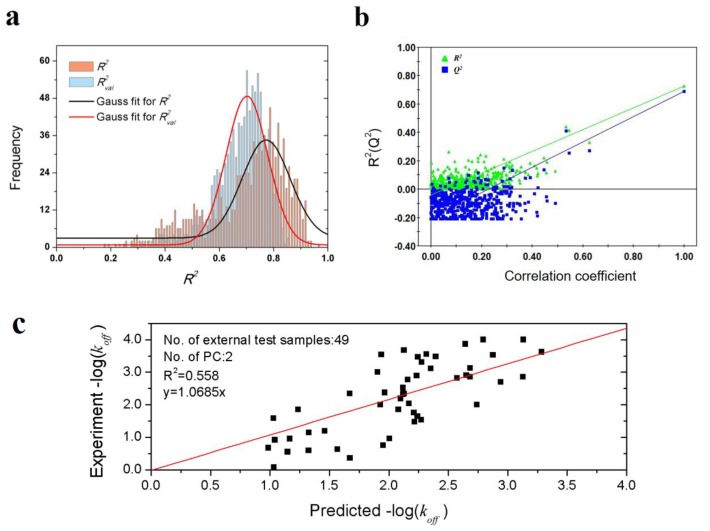
Results of PLS model validation. (**a**) *R*^2^ and *R^*2*^_val_* distributions of 1000-times repeated PLS modeling; (**b**) 500-times Y random permutation test; (**c**) scatter plot of experimental vs. predicted −log(*k_off_*) of 49 independent test samples.

To validate the robustness of the optimal PLS model, 1000-times repeated PLS modeling and 500-times Y-random permutation test were performed. Figure 3a shows the frequency distribution of *R*^2^ and *R^*2*^_val_* in 1000-times repeated PLS modeling based on the randomly selected training and validation samples. The means of *R*^2^ and *R^*2*^_val_* are 0.70 ± 0.15 and 0.67 ± 0.09, respectively. Besides, 500-times Y-random permutation test was also performed. From Figure 3b, it can be clearly observed that the *R*^2^ and *Q*^2^ drop sharply along with the decreased correlation coefficients between the original and permuted Y, which indicates a high-quality PLS model. To further test the predictive power of the optimal PLS model, 49 non-redundant HSP inhibitors (Table S3) were collected from Kokh’s research [10] as an independent test dataset. Although the *R^*2*^_val_* value was decreased in some degree, the performance is still acceptable for the independent test samples with different molecular skeletons (Table 2 and Figure 3c).

Table 2 shows the performance comparison among the available prediction models. Although the τRAMD and “COMBINE” models achieved satisfied prediction results, both of the models depend heavily on the energy-minimized structures of the ligand-receptor complexes and the results of which are hardly to be reproduced, which limits their real-life applications in a large degree.

### 2.2. Adenosine Receptor

ARs belongs to a class of G protein-coupled receptors (GPCR) and is responsible for regulating the physiological actions of adenosine [22]. Four AR subtypes have been found in humans, namely A_1_, A_2A_, A_2B_ and A_3_ [23]. Recent researches proposed that the agonists of A_1_AR contribute to the cardioprotection and immune regulation, while the antagonists can be used for asthma treatments. Herein, after removing the 7 molecules with fast dissociation rates (−log(*k_off_*) < 0.1), 27 A_1_AR agonists and 12 antagonists were derived from recent literatures [24,25,26,27,28] and used for constructing the prediction model of dissociation rate constant (Table S4). Herein, 26 molecules are randomly selected as training samples and remaining 13 as validation samples.

**Figure 4:**
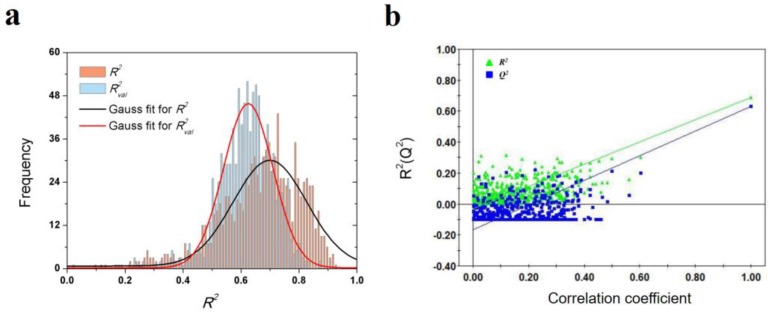
Results of PLS model validations. (**a**) frequency distribution of *R*^2^ and *R^*2*^_val_* in 1000-times repeated PLS modeling; (**b**) 500-times Y random permutation test.

As shown in Table 3, a total of 7 VolSurf descriptors were obtained from SMR feature selection. In consideration of the balanced performances on training and validation datasets, the PLS model with 2 descriptors was chosen as the optimal model, of which the *R*^2^, *Q*^2^ and *R^*2*^_val_* are 0.688, 0.631 and 0.627, respectively. The robustness and predictive power of the PLS model were further validated by 1000-times repeated PLS modeling and 500-times Y-random permutation test with excellent results obtained. The means of *R*^2^ and *R^*2*^_val_* for 1000-repeated PLS modeling are 0.66 ± 0.14 and 0.61 ± 0.10, respectively (Figure 4). The variables involved in the optimal PLS model are POL and W5-N3+, which indicates that the dissociation rate of A_1_AR ligands are closely related to molecular polarizability and hydrophilic interactions at −3.0 kcal/mol energy level.

**Figure 5:**
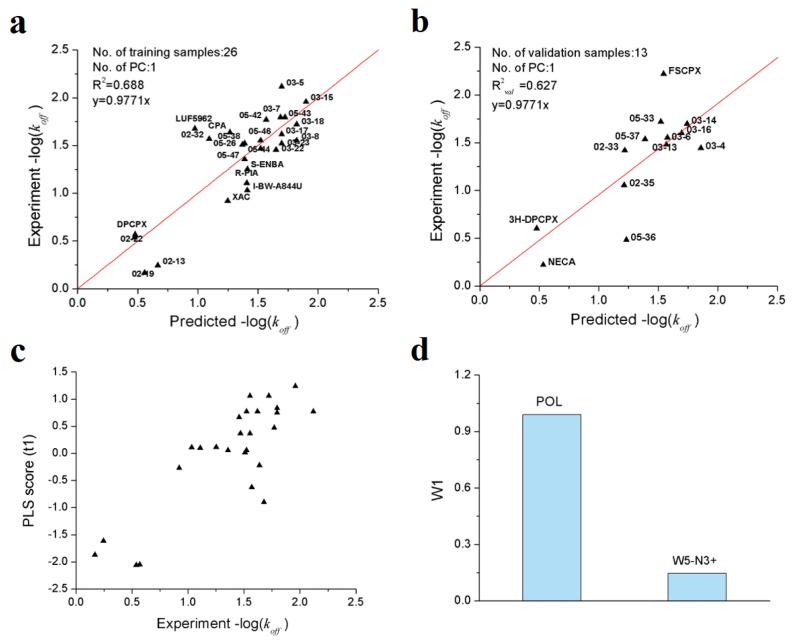
Optimal PLS model of the dissociation rate constants of 39 A1AR agonists/antagonists. (**a**) scatter plot of experimental vs. predicted −log(*k_off_*) of the 26 training samples; (**b**) scatter plot of experimental vs. predicted −log(*k_off_*) of the 13 validation samples; (**c**) first principle component scores of the 26 training samples; (**d**) weights of independent variables in the first principle component.

From Figure 5a,b, it is obvious that all the training/validation samples are distributed along the regression lines very well; that the first principal component scores are closely correlated with the observed −log(*k_off_*) values (Figure 5c). From the loading plot of POL and W5−N3+ variables (Figure 5d), it can be deduced that higher molecular polarizability and stronger hydrophilic interactions contribute to longer residence time.

### 2.3. p38 Mitogen-Activated Protein Kinase

As the key regulators of inflammatory cytokine expression, p38 MAPK plays an important role in a wide variety of essential physiological processes [29] and is closely associated with human diseases, such as asthma, autoimmunity and cancer, etc. [30,31]. Herein, 28 inhibitors of p38 MAPK (Table S5) with determined dissociation rate constants were collected from recent literatures [32,33,34,35], of which 18 were randomly chosen as training samples and 10 as validation samples.

**Figure 6:**
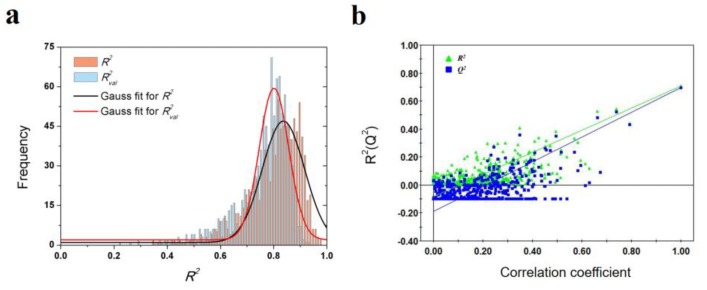
Validations of the optimal PLS model: (**a**) frequency distribution of *R*^2^ and *R^*2*^_val_* in 1000-times repeated PLS modeling; (**b**) 500-times Y random permutation test.

After variable selection and PLS modeling, an optimal PLS model with two variables was obtained, of which *R*^2^, *Q*^2^ and *R^*2*^_val_* for *k_off_* are 0.821, 0.818 and 0.821, respectively (Table 4). As showed in Table 5, it can be seen that the prediction performances of the VolSurf model is superior to that of the models based on position-restrained MD [15] and biased MD simulations [36]. Furthermore, the results of 1000-repeated PLS modeling and 500-times Y-random permutations test demonstrates that the high-quality PLS model is not caused by accident (Figure 6). The means of *R*^2^ and *R^*2*^_val_* for 1000-repeated PLS modeling are 0.80 ± 0.10 and 0.75 ± 0.10, respectively.

**Figure 7:**
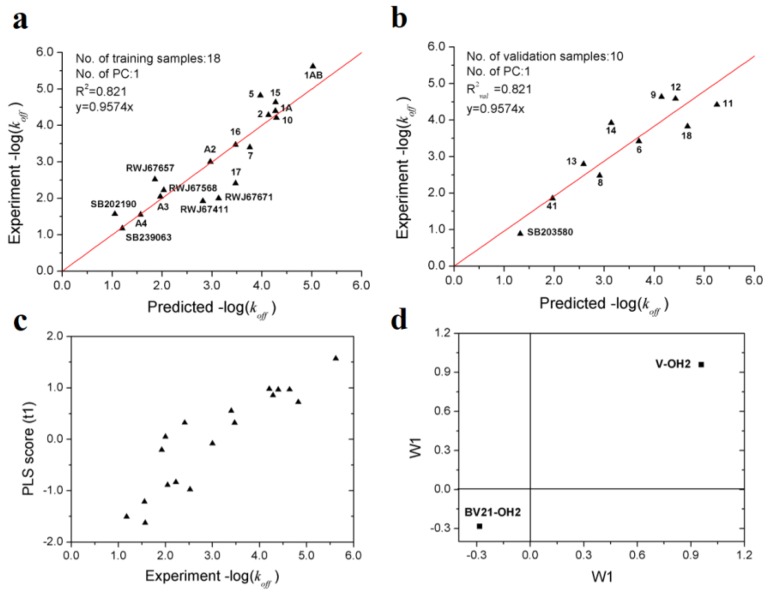
Optimal PLS model of the dissociation rate constants of 28 p38 MAPK inhibitors. (**a**) scatter plot of experimental vs. predicted −log(*k_off_*) of the 18 training samples; (**b**) scatter plot of experimental vs. predicted −log(*k_off_*) of the 10 validation samples; (**c**) first principle component scores of the 18 training samples. (**d**) weights of the two variables in the first principle component.

From Figure 7a,b, it can be inferred that the PLS model with two variables can accurately estimate the −log(*k_off_*) values which span about 5 orders of magnitude. The two descriptors (V-OH2 and BV21-OH2) selected imply that the dissociation rate of p38 MAPK inhibitors is mainly determined by molecular volume and the hydrophilic interactions at energy levels of −1.0 and −3.0 kcal/mol. From the PLS score plot (Figure 7c), it can be observed that the first principal component scores are significantly correlated with the observed −log(*k_off_*) values.

From the loading plot, it can be deduced that larger molecular volume contributes to longer residence time, while stronger hydrophilic interactions contribute to faster dissociation (Figure 7d). The result proposed in this study is consistent with previous researches, which suggested that type I kinases inhibitors bind to the ATP binding site and usually smaller and faster, while compounds of type II are generally large since occupy additional transient sub-pocket [36,37].

## 3. Methods

### 3.1. VolSurf

**Figure 8:**
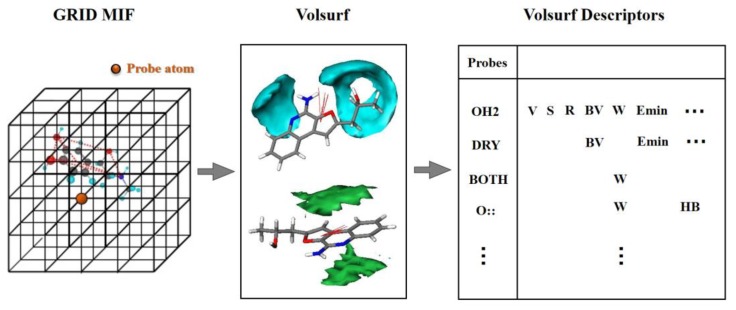
The procedure of VolSurf description: (1) dividing the molecular space into 3D lattice points; (2) calculating the interaction energies of molecules by specific probes in each lattice point; (3) quantitating the interaction volume and surface information. MIF: molecular interaction fields.

VolSurf [38,39] is a grid-based structural description method, which aims to calculate molecular properties from 3D molecular fields of interaction energies and compress most of the relevant information into few quantitative descriptors. As shown in Figure 8, VolSurf first divides molecular space into 3D lattice points. Subsequently, the interaction energies of the molecules with specific probes in each lattice point are calculated to acquire the interaction volume and surface information, which can quantitatively characterize the potential steric, electrostatic, H-bonding, hydrophobic, etc. interactions between the ligand and receptor [40].

VolSurf can build a unique framework related to specific molecular properties by using 9 probes, i.e., water probe (HO2), hydrophobic probe (DRY), carbonyl oxygen atom (O), amphipathic probe (BOTH), carboxy oxygen atom (O::), amide NH group (N1), sp^2^ N with one long pair (N:=), sp^3^ cationic NH3 group probe (N3+) and anionic phenolate oxygen atom (O-). In the last decade, VolSurf has been successfully used for predicting pharmacokinetics properties, i.e., absorption, distribution, metabolism and excretion properties [16,41,42].

Before VolSurf calculation, each molecule was first charged by MMFF94 method and then optimized by MMFF94 force field with conjugate gradient minimizer (Sybyl 8.1). The maximum iteration steps, energy gradient and long-distance cutoff were set to 5000 times, 0.05 kcal/mol·Å and 8 Å, respectively. A total of 166 VolSurf descriptors were generated by all 9 probes. In order to remove redundant variables, SMR was used for VolSurf feature selection, of which the entry and removal probabilities were set to 0.05 and 0.1. Then, the candidate variable subsets were employed for PLS modeling.

### 3.2. Partial Least Squares Regression

Partial least squares, developed by Herman O. A. Wold and Svante Wold [43], is a projection space-based statistical method that combines principal component analysis (PCA) [44] and multiple linear regression (MLR) and today widely used in the fields of chemometrics bioinformatics, sensometrics and neuroscience. In PLS, both the X and the Y variables are bilinearly decomposed and projected into a new principal component space (Equations (1) and (2)).

X = TP^T^ + E (1)

Y = UC^T^ + G (2)

where, T and P are the score and loading matrices of X; U and C are score and weight matrices of Y. E and G are the residual matrices. The aim of PLS is to find a linear relationship between X and Y, so that the scores of X matrix are good predictors of Y (Equation (3)),

Y = TC^T^ + F (3)

where F is the residual matrix of Y. *For* more details, please refer to references [45,46]. In this paper, the target variable (*k_off_*) was negative logarithm transformed before PLS modeling. Herein, the RMSE (Equation (4)) and MAPE (Equation (5)) were used for model validation, of which the *ŷ_i_* and *y_i_* represented the predicted and experimental −log(*k_off_*) values, respectively.

(4) $$\mathrm{RMSE}=\sqrt{\frac{1}{n}\overset{n}{\underset{i=n}{\sum }}{\left({\hat{y}}_{i}-y_{i}\right)}^{2}}$$

(5) $$\mathrm{MAPE}=\frac{1}{n}\overset{n}{\underset{i=1}{\sum }}\left|\frac{{\hat{y}}_{i}-y_{i}}{y_{i}}\right|$$

## 4. Conclusions

As we know, the 3D-grid-based VolSurf method has been specifically proposed to quantitatively predict pharmacokinetic properties, e.g., membrane permeation, transport and biotransformation, etc. However, in a recent research, we found that it can be also used for predicting the pharmacodynamics properties such as dissociation rate constants (*k_off_)*, which are also closely related to the molecular surface properties. In this study, we carried out in-depth studies by using the VolSurf method for predicting the ligand dissociation rate of heat shock protein 90α, A_1_AR and p38 mitogen-activated protein kinase. From the PLS modeling results, it can be concluded that few VolSurf descriptors can extract efficiently key molecular surface properties related to the dissociation rate, and the resulting PLS models are proved be robust and predictive. Although the MD-based and “COMBINE” strategies achieved equivalent or even better prediction performances, both of the strategies depend heavily on the energy-minimized structures of the ligand-receptor complexes and the results of which are hardly to be reproduced. By contrast, the VolSurf-based method requires only the information of chemical ligands and can provide fast and accurate predictions on the kinetic properties, which is extremely useful in virtual screening researches. However, due to the complexity in the ligand dissociation process, there are still a lot of questions that remain further researches. 

## Figures and Tables

**Table 1:** The partial least squares (PLS) modeling results of the dissociation rate constants of the Hsp90 inhibitors.

Model	No. of Variables	Variables Involved in Sequence *^b^*	No. of PCs	*R* ^2^	*Q* ^2 *c*^	RMSE *^d^*	MAPE *^e^*	*R^*2*^_val_*	RMSEP *^f^*	Equation
1	1	V-OH2	1	0.635	0.626	0.435	0.167	0.716	0.409	Y = 1.0727X
2 *^a^*	2	D8-DRY	2	0.726	0.688	0.377	0.145	0.718	0.404	Y = 1.0685X
3	3	W3-N3+	2	0.763	0.717	0.350	0.138	0.710	0.412	Y = 1.073X
4	4	Emin1-OH2	2	0.771	0.709	0.345	0.128	0.744	0.393	Y = 1.0753X
5	5	D4-DRY	2	0.768	0.707	0.346	0.125	0.758	0.387	Y = 1.0774X
6	6	A	2	0.788	0.695	0.331	0.161	0.766	0.399	Y = 1.0941X
7	7	IW8-OH2	2	0.815	0.726	0.310	0.186	0.780	0.393	Y = 1.0968X
8	8	W4-N:=	2	0.818	0.730	0.307	0.344	0.778	0.407	Y = 1.1073X
9	9	D13-DRY	2	0.819	0.658	0.306	0.143	0.730	0.437	Y = 1.1101X

*^a^* Optimal PLS model with two descriptors; *^b^* V-OH2: molecular volume given as the water solvent excluded volume (Å^3^); D8-DRY: hydrophobic regions generated by the hydrophobic probe at energy level of −1.6 kcal/mol; W3−N3+: hydrophilic regions generated by the sp3 NH3 probe at energy level of −1.0 kcal/mol; Emin1-OH2: local interaction energy minima between the H2O probe and the target molecule; D4-DRY: hydrophobic regions generated by the hydrophobic probe at energy level of −0.8 kcal/mol; A: Amphiphilic moment, defined as a vector pointing from the center of the hydrophobic domain to the center of the hydrophilic domain; IW8-OH2: integy moments generated by the water probe at energy level of −6.0 kcal/mol, represent the unbalance between the center of mass of a molecule and the position of the hydrophilic regions around it; W4-N:=: hydrophilic regions generated by the sp^2^ N probe at energy level of −2.0 kcal/mol; D13-DRY: hydrophobic local interaction energy minima distances generated by the hydrophobic probe; *^c^* 5-fold cross validation; *^d^* RMSE: Root- mean-square error of prediction for training samples; *^e^* MAPE: Mean absolute percentage error for training samples; *^f^* RMSEP: RMSE for validation samples.

**Table 2:** Performance comparison among VolSurf, τ-random acceleration molecular dynamics (τRAMD) and COMBINE models.

Model	Need to Consider Receptors?	No. of Variables	Total Sample Size	Training/Validation/Test Samples	*R* ^2^	*Q* ^2^	MAPE	*R^*2*^_val_*	*R^*2*^_test_*
VolSurf	No	2	101	35/17/49	0.73	0.69 *^a^*	0.15	0.72	0.56
τRAMD [10]	Yes	NA	70*^c^*	59/0/0	0.66	NA	NA	NA	NA
τRAMD [11]	Yes	NA	94*^d^*	80/0/0	0.75	NA	0.39	NA	NA
COMBINE [12]	Yes	42	70 *^e^*	53/13/0	0.80	0.69 *^b^*	0.37	0.86	NA

*R*^2^: Coefficient of determination; *Q*^2^: the cross-validated *R*^2^; *R^*2*^_val_*: *R*^2^ for validation samples; *R^*2*^_test_*: *R*^2^ for external test samples; *^a^* 5-fold cross-validation; *^b^* leave-one-out cross-validation; *^c^* 11 samples removed as outliers; *^d^* 14 samples removed as outliers; *^e^* 4 samples removed as outliers.

**Table 3:** PLS modeling results of the dissociation rate constants of 39 A1AR agonists/antagonists.

Model	No. of Variables	Variables Involved in Sequence *^b^*	No. of PCs	*R* ^2^	*Q^2 c^*	RMSE	MAPE	*R^*2*^_val_*	RMSEP	Equation
1	1	POL	1	0.638	0.606	0.299	0.201	0.584	0.341	Y = 0.9691X
2 *^a^*	2	W5-N3+	1	0.688	0.631	0.278	0.201	0.627	0.284	Y = 0.9771X
3	3	W2-O	2	0.644	0.549	0.297	0.197	0.581	0.290	Y = 0.9873X
4	4	Emin2-DRY	2	0.678	0.599	0.282	0.193	0.489	0.311	Y = 0.9814X
5	5	D13-OH2	2	0.717	0.623	0.264	0.185	0.561	0.277	Y = 0.9912X
6	6	BV21-DRY	2	0.757	0.610	0.245	0.165	0.579	0.279	Y = 0.9934X
7	7	ID1-DRY	2	0.762	0.600	0.242	0.150	0.647	0.257	Y = 1.0237X

*^a^* Optimal PLS model with 2 descriptors; *^b^* POL: average molecular polarizability; W5-N3+: hydrophilic regions generated by the sp3 NH3 probe at energy level of −3.0 kcal/mol (More details please refer to Table S1); *^c^* leave-one-out cross-validation.

**Table 4:** PLS modeling results of the dissociation rate constants of p38 mitogen-activated protein kinase (p38 MAPK) inhibitors.

Model	No. of Variables	Variables Involved in Sequence *^b^*	No. of PCs	*R* ^2^	*Q* ^2 *c*^	RMSE	MAPE	*R^*2*^_val_*	RMSEP	Equation
1	1	V-OH2	1	0.709	0.696	0.696	0.192	0.685	0.671	Y = 0.9862X
2 *^a^*	2	BV21-OH2	1	0.821	0.818	0.546	0.145	0.821	0.527	Y = 0.9574X
3	3	IW3-OH2	1	0.881	0.882	0.445	0.122	0.713	0.683	Y = 0.9344X
4	4	Emin1-OH2	1	0.877	0.856	0.453	0.159	0.604	0.765	Y = 0.9579X
5	5	W8	1	0.821	0.808	0.546	0.160	0.628	0.727	Y = 0.993X
6	6	D7-DRY	1	0.868	0.827	0.469	0.130	0.567	0.800	Y = 0.9561X
7	7	D6-DRY	1	0.853	0.760	0.465	0.135	0.481	0.905	Y = 0.9224X
8	8	W8-O	1	0.846	0.751	0.506	0.138	0.484	0.895	Y = 0.9282X
9	9	IW7-OH2	1	0.859	0.756	0.485	0.125	0.490	0.905	Y = 0.9167X

*^a^* Optimal PLS model with two descriptors; *^b^* V-OH2: molecular volume given as the water solvent excluded volume (Å^3^); BV21-OH2: the best hydrophilic volumes generated by the water probe at energy levels of −1.0 and −3.0 kcal/mol. (More details please refer to Table S1); *^c^* 3-fold cross validation.

**Table 5:** Performance comparison among VolSurf, position-restrained and biased MD models.

Model	Need to Consider Receptors?	No. of Variables	Total Sample Size	Training/Validation Samples	*R* ^2^	*Q* ^2^	*R^*2*^_val_*
VolSurf	No	2	28	18/10	0.82	0.82 *^a^*	0.82
Position-restrained MD [15]	Yes	3	20	14/6	0.72	0.66 *^b^*	0.56
Biased MD [36]	Yes	NA	8	8/0	0.64	NA	NA

*^a^* 3-fold cross-validation; *^b^* leave-one-out cross-validation.

## Supplementary Materials

The following are available online at https://www.mdpi.com/1422-0067/21/7/2456/s1, Table S1: The definitions of the involved VolSurf variables, Table S2: The *k_off_* values of 52 HSP inhibitors, Table S3: The *k_off_* values of 49 non-redundant HSP inhibitors, Table S4: The *k_off_* values of 46 A_1_AR ligands, Table S5: The *k_off_* values of 28 p38 MAPK inhibitors.
