# Supplementary material for: In Silico Prediction of the Dissociation Rate Constants of Small Chemical Ligands by 3D-Grid-Based VolSurf Method
Source: Int J Mol Sci. 2020 Apr 2;21(7):2456. doi: 10.3390/ijms21072456 (PMC7177943; doi:10.3390/ijms21072456)
Supplement: Supplementary file 1 [file ijms-21-02456-s001.pdf]

Supplementary Material

In silico Prediction of the Dissociation Rate Constants of Small Chemical Ligands by 3D-grid-based VolSurf Method

Shuheng Huang[1,2,†], Linxin Chen[1,2,†], Hu Mei[1,2,*], Duo Zhang[2], Tingting Shi[2], Zuyin Kuang[2], Yu Heng[2], Lei Xu[2], Xianchao Pan[2,3,*]

[1] Key Laboratory of Biorheological Science and Technology (Ministry of Education), Chongqing University, Chongqing 400044, China

[2] College of Bioengineering, Chongqing University, Chongqing 400044, China

[3] Department of Medicinal Chemistry, College of Pharmacy, Southwest Medical University, Luzhou, Sichuan, 646000, China

Table of contents

	Contents	Page
Table S1	The definitions of the involved VolSurf variables	S2-3
Table S2	The k_{off} values of 52 HSP inhibitors	S4-5
Table S3	The k_{off} values of 49 non-redundant HSP inhibitors	S6-7
Table S4	The k_{off} values of 46 A_1AR ligands	S8-9
Table S5	The k_{off} values of 28 p38 MAPK inhibitors	S10

Table S1. The definitions of the involved VolSurf variables

Case	No.	Variables	Detail meaning
Heat shock protein 90α (HSP90)	1	V-OH2	Molecular volume given as the water solvent excluded volume (Å^3)
	2	D8-DRY	Hydrophobic regions generated by the hydrophobic probe at energy level of -1.6 kcal/mol
	3	W3-N3+	Hydrophilic regions generated by the sp3 NH3 probe at energy level of -1.0 kcal/mol
	4	Emin1-OH2	Local interaction energy minima between the H2O probe and the target molecule
	5	D4-DRY	Hydrophobic regions generated by the hydrophobic probe at energy level of -0.8 kcal/mol
	6	A	Amphiphilic Moment, defined as a vector pointing from the center of the hydrophobic domain to the center of the hydrophilic domain
	7	IW8-OH2	Integy moments generated by the water probe at energy level of -6.0 kcal/mol, represent the unbalance between the center of mass of a molecule and the position of the hydrophilic regions around it
	8	W4-N:=	Hydrophilic regions generated by the sp^2 N probe at energy level of -2.0 kcal/mol
	9	D13-DRY	Hydrophobic local interaction energy minima distances generated by the hydrophobic probe
Adenosine receptor (A_1AR)	1	POL	The average molecular polarizability
	2	W5-N3+	Hydrophilic regions generated by the sp3 NH3 probe at energy level of -3.0 kcal/mol
	3	W2-O	Hydrophilic regions generated by the carbonyl oxygen atom at energy level of -0.5 kcal/mol
	4	Emin2-DRY	The second local minima of interaction energy between the DRY probe and the target molecule
	5	D13-OH2	Hydrophobic local interaction energy minima distances generated by the water probe
	6	BV21-DRY	The best hydrophobic volumes generated by the hydrophobic probe at energy levels of -1.0 and -3.0 kcal/mol
	7	ID1-DRY	The distances between the molecular center of mass and the barycenter of the hydrophobic interaction region calculated from

			hydrophobic probe at energy level of -0.2 kcal/mol
p38 mitogen-activated protein kinase (p38 MAPK)	1	V-OH2	Molecular volume given as the water solvent excluded volume (Å^3)
	2	BV21-OH2	The best hydrophilic volumes generated by the water probe at energy levels of -1.0 and -3.0 kcal/mol
	3	IW3-OH2	Integy moments generated by the water probe at energy level of -1.0 kcal/mol, represent the unbalance between the center of mass of a molecule and the position of the hydrophilic regions around it
	4	Emin1-OH2	Local interaction energy minima between the H2O probe and the target molecule
	5	W8	The hydrophilic regions, represent the molecular envelope accessible generated by solvent water probe at energy level of -6.0 kcal/mol
	6	D7-DRY	Hydrophobic regions generated by the hydrophobic probe at energy level of -1.4 kcal/mol
	7	D6-DRY	Hydrophobic regions generated by the hydrophobic probe at energy level of -1.2 kcal/mol
	8	W8-O	The hydrophilic regions, represent the molecular envelope accessible generated by the carbonyl oxygen atom at energy level of -6.0 kcal/mol
	9	IW7-OH2	Integy moments generated by the water probe at energy level of -5.0 kcal/mol, represent the unbalance between the center of mass of a molecule and the position of the hydrophilic regions around it

Table S2. The k_{off} values of 52 Hsp90 inhibitors

Class	Compound	k_{off} (s^{-1})	-log(k_{off})	Smiles
Cavity-varying indazoles	1a	2.90×10^{-3}	2.5376	CCCc1n[nH]c2cc(O)c(C(=O)N(C)c3ccc(N4CCOCC4)cc3)cc12
	1b	2.70×10^{-3}	2.5686	CCCCc1n[nH]c2cc(O)c(C(=O)N(C)c3ccc(N4CCOCC4)cc3)cc12
	1c	1.61×10^{-3}	2.7932	CCC(C)c1n[nH]c2cc(O)c(C(=O)N(C)c3ccc(N4CCOCC4)cc3)cc12
	1d	3.05×10^{-3}	2.5157	COCc1n[nH]c2cc(O)c(C(=O)N(C)c3ccc(N4CCOCC4)cc3)cc12
	1e	2.90×10^{-1}	0.5376	CCC(=O)c1n[nH]c2cc(O)c(C(=O)N(C)c3ccc(N4CCOCC4)cc3)cc12
	1f	2.34×10^{-2}	1.6308	CCC(O)c1n[nH]c2cc(O)c(C(=O)N(C)c3ccc(N4CCOCC4)cc3)cc12
	1g	6.79×10^{-4}	3.1681	Cc1cccc(Cc2n[nH]c3cc(O)c(C(=O)N(C)c4ccc(N5CCOCC5)cc4)cc23)c1
	1h	2.60×10^{-3}	2.585	COc1cccc(Cc2n[nH]c3cc(O)c(C(=O)N(C)c4ccc(N5CCOCC5)cc4)cc23)c1
	1i	1.35×10^{-3}	2.8697	CC(C)CN(C)C(=O)c1[nH]nc2cc(O)c(C(=O)N(C)c3ccc(N4CCOCC4)cc3)cc12
	1j	1.32×10^{-2}	1.8794	CN(C(=O)c1cc2c(C(=O)N3CCOCC3)n[nH]c2cc1O)c1ccc(N2CCOCC2)cc1
	3a	2.80×10^{-3}	2.5528	CN(C(=O)c1cc2c(CC3CCCCC3)n[nH]c2cc1O)c1ccc(N2CCOCC2)cc1
Cavity-varying resorcinoles	4a	2.63×10^{-3}	2.58	Cc1ccccc1-n1c(-c2cc(C(=O)N(C)Cc3ccccc3-c3cccnc3)c(O)cc2O)n[nH]c1=O
	4b	1.12×10^{-3}	2.9508	Cc1ccccc1-n1c(-c2cc(C(=O)N(C)Cc3ccccc3)c(O)cc2O)n[nH]c1=O
	4c	1.07×10^{-3}	2.9706	Cc1ccccc1CN(C)C(=O)c1cc(-c2n[nH]c(=O)n2-c2ccccc2C)c(O)cc1O
	4d	3.06×10^{-3}	2.5143	Cc1ccccc1-n1c(-c2cc(C(=O)N(C)Cc3cccc(-c4cncnc4)c3)c(O)cc2O)n[nH]c1=O
	4e	4.72×10^{-3}	2.3261	COc1ccc(CN(C)C(=O)c2cc(-c3n[nH]c(=O)n3-c3ccccc3C)c(O)cc2O)cc1
	4f	1.25×10^{-3}	2.9031	COc1ccccc1CN(C)C(=O)c1cc(-c2n[nH]c(=O)n2-c2ccccc2C)c(O)cc1O
	4g	9.85×10^{-4}	3.0066	Cc1cccc(CN(C)C(=O)c2cc(-c3n[nH]c(=O)n3-c3ccccc3C)c(O)cc2O)c1
	4h	6.53×10^{-3}	2.1851	Cc1ccccc1-n1c(-c2cc(C(=O)N(C)CC3CCCO3)c(O)cc2O)n[nH]c1=O
	4i	1.79×10^{-3}	2.7471	Cc1ccccc1-n1c(-c2cc(C(=O)N(C)Cc3cccs3)c(O)cc2O)n[nH]c1=O
Entrance-varying indazoles	5a	1.61×10^{-2}	1.7932	COc1cccc(N(C)C(=O)c2cc3c(Cc4cccc(C)c4)n[nH]c3cc2O)c1
	5b	1.17×10^{-2}	1.9318	Cc1cccc(Cc2n[nH]c3cc(O)c(C(=O)N(C)c4cccc(Cl)c4)cc23)c1
	5c	2.95×10^{-3}	2.5302	Cc1cccc(Cc2n[nH]c3cc(O)c(C(=O)N(C)c4ccc(CN5CCOCC5)cc4)cc23)c1
	5d	3.00×10^{-3}	2.5229	Cc1cccc(Cc2n[nH]c3cc(O)c(C(=O)N(C)c4ccc(OCCN(C)C)cc4)cc23)c1
	5e	9.13×10^{-3}	2.0395	Cc1cccc(Cc2n[nH]c3cc(O)c(C(=O)N(C)c4ccccc4)cc23)c1
	5f	4.00×10^{-3}	2.3979	COc1ccc(N(C)C(=O)c2cc3c(Cc4cccc(C)c4)n[nH]c3cc2O)cc1
	5g	1.43×10^{-2}	1.8447	Cc1cccc(Cc2n[nH]c3cc(O)c(C(=O)N(C)c4ccc(Cl)cc4)cc23)c1
	5h	7.52×10^{-4}	3.1238	Cc1cccc(Cc2n[nH]c3cc(O)c(C(=O)N(C)c4ccc(N5CCN(C)CC5)cc4)cc23)c1
	5i	2.09×10^{-3}	2.6799	Cc1cccc(Cc2n[nH]c3cc(O)c(C(=O)N(C)c4ccc(OCCCC#N)cc4)cc23)c1
	5j	5.03×10^{-3}	2.2984	Cc1ccc(N(C)C(=O)c2cc3c(Cc4cccc(C)c4)n[nH]c3cc2O)cc1
	5k	2.95×10^{-3}	2.5302	COc1ccc(N(C)C(=O)c2cc3c(Cc4cccc(C)c4)n[nH]c3cc2O)cc1Cl
	5l	1.08×10^{-2}	1.9666	Cc1cccc(Cc2n[nH]c3cc(O)c(C(=O)N(C)c4ccc(Br)cc4)cc23)c1
	5m	1.24×10^{-3}	2.9066	Cc1cccc(Cc2n[nH]c3cc(O)c(C(=O)N(C)c4ccc(N5CCNCC5)cc4)cc23)c1
	5n	1.37×10^{-3}	2.8633	Cc1cccc(Cc2n[nH]c3cc(O)c(C(=O)N(C)c4ccc(N5CCOCC5=O)cc4)cc23)c1
	5o	2.02×10^{-2}	1.6946	Cc1cccc(Cc2n[nH]c3cc(O)c(C(=O)N(C)c4cccc(F)c4)cc23)c1

	5p	2.37×10^{-3}	2.6253	Cc1cccc(Cc2n[nH]c3cc(O)c(C(=O)N(C)c4ccc(N(C)C)cc4)cc23)c1
	5q	8.90×10^{-3}	2.0506	CC(=O)c1ccc(N(C)C(=O)c2cc3c(Cc4cccc(C)c4)n[nH]c3cc2O)cc1
	5r	5.00×10^{-3}	2.301	Cc1cccc(Cc2n[nH]c3cc(O)c(C(=O)N(C)c4ccc5c(c4)OCO5)cc23)c1
	5s	4.34×10^{-3}	2.3625	Cc1cccc(Cc2n[nH]c3cc(O)c(C(=O)N(C)c4ccc(CNS(C)(=O)=O)cc4)cc23)c1
	5t	3.18×10^{-2}	1.4976	Cc1cccc(Cc2n[nH]c3cc(O)c(C(=O)N(C)c4ccc(F)cc4)cc23)c1
	5u	1.26×10^{-2}	1.8996	CC(=O)N1CCc2cc(N(C)C(=O)c3cc4c(Cc5cccc(C)c5)n[nH]c4cc3O)ccc21
	5v	7.70×10^{-3}	2.1135	Cc1cccc(Cc2n[nH]c3cc(O)c(C(=O)N(C)c4cccc(C)c4)cc23)c1
	5w	6.31×10^{-3}	2.2	Cc1cccc(Cc2n[nH]c3cc(O)c(C(=O)N(C)c4ccc5c(c4)OCCO5)cc23)c1
	5x	5.63×10^{-3}	2.2495	CC(=O)N1CCc2ccc(N(C)C(=O)c3cc4c(Cc5cccc(C)c5)n[nH]c4cc3O)cc21
Prospective indazole	2a	1.60×10^{-3}	2.7959	CN(C(=O)c1cc2c(CC3CCCC3)n[nH]c2cc1O)c1ccc(N2CCOCC2)cc1
	3b	9.10×10^{-4}	3.041	CN(C(=O)c1cc2c(C(=O)N3CCCCC3)n[nH]c2cc1O)c1ccc(N2CCOCC2)cc1
	3c	2.94×10^{-4}	3.5317	COC1CCCN(C(=O)c2n[nH]c3cc(O)c(C(=O)N(C)c4ccc(N5CCOCC5)cc4)cc23)C1
	2b	2.43×10^{-3}	2.6144	CN(C(=O)c1cc2c(C(=O)N3CCCC3)n[nH]c2cc1O)c1ccc(N2CCOCC2)cc1
	2c	1.38×10^{-3}	2.8601	COC1CCN(C(=O)c2n[nH]c3cc(O)c(C(=O)N(C)c4ccc(N5CCOCC5)cc4)cc23)C1
Quinazolines	6a	5.52×10^{-1}	0.2581	Nc1nc(C(=O)N2Cc3ccccc3C2)c2ccccc2n1
	6b	5.73×10^{-1}	0.2418	Nc1nc(C(=O)N2Cc3ccccc3C2)c2cc(O)ccc2n1
	6c	5.54×10^{-1}	0.2565	Nc1nc(C(=O)N2Cc3ccc(O)cc3C2)c2ccccc2n1

Table S3. The k_{off} values of 49 non-redundant HSP inhibitors

ID	k_{off} (s^{-1})	-log(k_{off})	Smiles
1	1.00×10^{-4}	4	CCNC(=O)c1noc(-c2cc(C(C)C)c(O)cc2O)c1-c1ccc(C[NH+]2CCOCC2)cc1
2	2.10×10^{-4}	3.6778	CCNC(=O)c1noc(-c2cc(Cl)c(O)cc2O)c1-c1ccc(OC)cc1
3	1.00×10-2	2	CCNC(=O)c1[nH]nc(-c2cc(Cl)c(O)cc2O)c1-c1ccc(OC)cc1
4	1.00×10^{-4}	4	CCNC(=O)c1noc(c2cc(Cl)c(O)cc2O)c1c3ccc(C[NH+]4CCOCC4)cc3
5	1.40×10^{-2}	1.8539	O=c1[nH]nc(-c2cc(Br)c(O)cc2O)n1-c1ccccc1F
6	1.09×10^{-1}	0.9626	COc1ccc(-c2c(C#N)c(N)nc3sc(C(N)=O)c(N)c23)cc1OCCCC(=O)O
7	6.34×10^{-2}	1.1979	CCc1cc(-c2n[nH]c(C)c2-c2ccccc2F)c(O)cc1O
8	2.10×10^{-1}	0.6778	O=c1[nH]nc(-c2ccc(O)cc2O)n1-c1ccccc1F
9	8.25×10^{-1}	0.0835	Cc1n[nH]c2cc(O)c(-c3ccnn3-c3ccccc3)cc12
10	2.54×10^{-1}	0.5952	COc1ccc(-c2c(-c3ccc(O)cc3O)n[nH]c2C)cc1
11	3.30×10^{-4}	3.4815	CN(Cc1ccco1)C(=O)c1cc(-c2n[nH]c(=O)n2-c2ccccc2F)c(O)cc1O
13	1.70×10^{-3}	2.7696	CCCCN(C)C(=O)c1cc(-c2n[nH]c(=O)n2-c2ccccc2F)c(O)cc1O
14	1.74×10^{-1}	0.7595	Oc1cc(O)c(-c2ccnn2-c2ccccc2Cl)cc1CCc1ccccn1
15	6.40×10^{-3}	2.1938	CCCN(C)S(=O)(=O)c1cc(-c2n[nH]c(=O)n2-c2ccccc2F)c(O)cc1O
16	1.40×10^{-2}	1.8539	CC(C)N(C)S(=O)(=O)c1cc(-c2n[nH]c(=O)n2-c2ccccc2F)c(O)cc1O
17	2.78×10^{-4}	3.556	Brc1cnc2[nH]cnc2c1C(=O)NC1c2ccccc2-c2c(-c3cnc4ccccc4c3)cccc21
19	2.85×10^{-4}	3.5452	O=C(NC1c2ccccc2-c2c(-c3nc4ccncc4[nH]3)cccc21)c1ccnc2[nH]ccc12
20	1.36×10-4	3.8665	Cc1nn(-c2ccc(C(N)=O)c(N[C@H]3CC[C@H](O)CC3)c2)c2cccc(-c3cnc4ccccc4c3)c12
21	4.85×10^{-4}	3.3143	Cc1cn(-c2ccc(C(N)=O)c(N[C@H]3CC[C@H](O)CC3)c2)c2c1C(=O)CC(C)(C)C2
22	7.65×10^{-4}	3.1163	Cc1cn(-c2ccc(C(N)=O)c(NC3CCC(=O)CC3)c2)c2c1C(=O)CC(C)(C)C2
23	9.06×10^{-3}	2.0429	CC(C)N(C)S(=O)(=O)c1cc(-c2n[nH]c(=O)n2-c2ccccc2Cl)c(O)cc1O
24	4.70×10^{-3}	2.3279	CCCN(C)C(=O)c1cc(-c2n[nH]c(=O)n2-c2ccccc2C)c(O)cc1O
26	1.00×10^{-2}	2	CCCCCCN(C)C(=O)c1cc(-c2ccnn2-c2ccccc2C)c(O)cc1O
29	3.00×10^{-3}	2.5229	CCCCN(C)C(=O)c1cc(-c2n[nH]c(=O)n2-c2ccccc2)c(O)cc1O
30	3.40×10^{-4}	3.4685	Cc1ccccc1-n1nccc1-c1cc(C(=O)N(C)Cc2ccco2)c(O)cc1O
31	1.10×10^{-1}	0.9586	Cc1ccccc1-n1c(-c2ccc(O)cc2O)n[nH]c1=O
32	1.20×10^{-1}	0.9208	O=c1[nH]nc(-c2ccc(O)cc2O)n1-c1ccccc1Cl
33	7.10×10^{-2}	1.1487	CCc1ccccc1-n1c(-c2ccc(O)cc2O)n[nH]c1=O
34	4.20×10^{-3}	2.3768	CCCN(C)C(=O)c1cc(-c2n[nH]c(=O)n2-c2ccccc2F)c(O)cc1O
36	1.39×10^{-3}	2.857	C[NH+]1CCC(c2ccc(N(C)C(=O)c3cc4c(CCC(C)(C)C)n[nH]c4cc3O)cc2)CC1
37	2.01×10^{-3}	2.6968	CCCCN(C)C(=O)c1n[nH]c2cc(O)c(C(=O)N(C)c3ccc(N4CCOCC4)cc3)cc12
38	2.88×10^{-2}	1.5406	CN(Cc1ccc(Cl)cc1)C(=O)c2cc3c(Cc4ccccc4)n[nH]c3cc2O
39	2.24×10^{-2}	1.6498	Cc1cccc(Cc2n[nH]c3cc(O)c(C(=O)N(C)Cc4ccccc4)cc23)c1
40	1.74×10^{-2}	1.7595	Cc1cccc(Cc2n[nH]c3cc(O)c(C(=O)N(C)Cc4ccc(Cl)cc4)cc23)c1
41	2.32×10^{-1}	0.6345	Oc1cc2[nH]nc(Cc3ccccc3)c2cc1-c1ccnn1-c1ccccc1
45	7.42×10^{-4}	3.1296	Cc1cccc(Cc2n[nH]c3cc(O)c(C(=O)N(C)c4ccc(N5CCCCC5)cc4)cc23)c1
47	1.24×10^{-3}	2.9066	Cc1cccc(Cc2n[nH]c3cc(O)c(C(=O)N(C)c4ccc(N5CC[NH2+]CC5)cc4)cc23)c1
49	1.38×10^{-3}	2.8601	CO[C@H]1CCN(C(=O)c2n[nH]c3cc(O)c(C(=O)N(C)c4ccc(N5CCOCC5)cc4)cc23)C1
50	2.94×10^{-4}	3.5317	CO[C@H]1CCCN(C(=O)c2n[nH]c3cc(O)c(C(=O)N(C)c4ccc(N5CCOCC5)cc4)cc23)C1
60	2.38×10^{-4}	3.6234	C[NH+]1CCN(S(=O)(=O)c2ccccc2-c2ccc3nc(N)nc(C(=O)N4Cc5ccccc5C4)c3c2)CC1

61	2.79×10^{-1}	0.5544	Cc1ccc2nc(N)nc(C(=O)N3Cc4ccccc4C3)c2c1
62	4.53×10^{-3}	2.3439	CNCc1ccccc1-c1ccc2nc(N)nc(C(=O)N3Cc4ccccc4C3)c2c1
63	1.51×10^{-3}	2.821	Nc1nc(C(=O)N2Cc3ccccc3C2)c2cc(-c3cc(F)c(F)cc3CCc3nnn[nH]3)ccc2n1
65	4.54×10^{-3}	2.3429	Nc1nc(C(=O)N2Cc3ccccc3C2)c2cc(-c3ccccc3O)ccc2n1
66	1.27×10^{-3}	2.8962	COc1c(C)cnc(Cn2cc(C#CCC(C)(C)O)c3c(Cl)nc(N)nc32)c1C
67	2.56×10^{-2}	1.5918	Cc1cnc(Cn2ccc3c(Cl)nc(N)nc32)c(C)c1Cl
68	3.31×10^{-2}	1.4802	C#CCCCn1c(Cc2cc(OC)c(OC)c(OC)c2Cl)nc2c(N)nc(F)nc21
69	4.29×10^{-1}	0.3675	N#Cc1ccc(N2CCN(CCCc3c[nH]c4cc(O)c(C#N)cc34)CC2)cc1
70	9.89×10^{-4}	3.0048	Nc1cc(C(=O)NC2c3ccccc3-c3c(-c4nc5ccncc5[nH]4)cccc32)ccn1

Table S4. The k_{off} values of 46 A_1AR ligands

Class	ID	k_{off} (min^{-1})	-log(k_{off})	Smiles
Agonist	03-13	3.30×10^{-2}	1.4815	COc1ccc(-c2c(C#N)c(N)nc(SCc3csc(-c4ccc(F)cc4)n3)c2C#N)cc1
	03-14	2.00×10^{-2}	1.699	COc1ccc(-c2c(C#N)c(N)nc(SCc3csc(-c4ccc(Br)cc4)n3)c2C#N)cc1
	03-15	1.10×10^{-2}	1.9586	COc1ccc(-c2c(C#N)c(N)nc(SCc3csc(-c4ccc(I)cc4)n3)c2C#N)cc1
	03-16	2.50×10^{-2}	1.6021	COc1ccc(-c2c(C#N)c(N)nc(SCc3csc(-c4cccc(Cl)c4)n3)c2C#N)cc1
	03-17	2.40×10^{-2}	1.6198	COc1ccc(-c2c(C#N)c(N)nc(SCc3csc(-c4ccccc4Cl)n3)c2C#N)cc1
	03-18	1.90×10^{-2}	1.7212	COc1ccc(-c2c(C#N)c(N)nc(SCc3csc(-c4ccc(Cl)c(Cl)c4)n3)c2C#N)cc1
	03-22	3.50×10^{-2}	1.4559	COc1ccc(-c2c(C#N)c(N)nc(SCc3csc(-c4ccc(Cl)cn4)n3)c2C#N)cc1
	03-23	3.00×10^{-2}	1.5229	COc1ccc(-c2c(C#N)c(N)nc(SCc3csc(-c4ccc(Br)cn4)n3)c2C#N)cc1
	03-4	3.60×10^{-2}	1.4437	N#Cc1c(N)nc(SCc2csc(-c3ccc(Cl)cc3)n2)c(C#N)c1-c1ccc(OCCO)cc1
	03-5	7.60×10^{-3}	2.1192	COc1ccc(-c2c(C#N)c(N)nc(SCc3csc(-c4ccc(Cl)cc4)n3)c2C#N)cc1
	03-6	2.80×10^{-2}	1.5528	N#Cc1c(N)nc(SCc2csc(-c3ccc(Cl)cc3)n2)c(C#N)c1-c1ccc(O)cc1
	03-7	1.60×10^{-2}	1.7959	N#Cc1c(N)nc(SCc2csc(-c3ccc(Cl)cc3)n2)c(C#N)c1-c1ccc2c(c1)OCO2
	03-8	2.80×10^{-2}	1.5528	COc1ccc(-c2c(C#N)c(N)nc(SCc3nc(-c4ccc(Cl)cc4)sc3Cl)c2C#N)cc1
	05-42	1.70×10^{-2}	1.7696	N#Cc1c(N)nc(SCc2csc(-c3ccc(Br)cc3)n2)nc1-c1ccc2c(c1)OCO2
	05-44	3.40×10^{-2}	1.4685	N#Cc1c(N)nc(SCc2csc(-c3cccc(Cl)c3)n2)nc1-c1ccc2c(c1)OCO2
	05-26	3.10×10^{-2}	1.5086	N#Cc1c(N)nc(SCc2csc(-c3ccc(Cl)cc3)n2)nc1-c1ccccc1
	05-33	1.90×10^{-2}	1.7212	N#Cc1c(N)nc(SCc2csc(-c3ccc(Cl)cc3)n2)nc1-c1ccc2c(c1)OCO2
	05-36	3.28×10^{-1}	0.4841	N#Cc1c(N)nc(SCc2coc(-c3cccc(F)c3)n2)nc1-c1ccc2c(c1)OCO2
	05-37	2.90×10^{-2}	1.5376	N#Cc1c(N)nc(SCc2csc(-c3ccc(F)cc3)n2)nc1-c1ccc2c(c1)OCO2
	05-38	3.00×10^{-2}	1.5229	N#Cc1c(N)nc(SCc2csc(-c3cccc(F)c3)n2)nc1-c1ccc2c(c1)OCO2
	05-43	1.60×10^{-2}	1.7959	N#Cc1c(N)nc(SCc2csc(-c3ccc(I)cc3)n2)nc1-c1ccc2c(c1)OCO2
	05-46	2.80×10^{-2}	1.5528	N#Cc1c(N)nc(SCc2csc(-c3ccccc3Cl)n2)nc1-c1ccc2c(c1)OCO2
	05-47	4.40×10^{-2}	1.3565	N#Cc1c(N)nc(SCc2csc(-c3ccccc3F)n2)nc1-c1ccc2c(c1)OCO2
	05-52*	8.70×10^{-1}	0.0605	N#Cc1c(N)nc(SCc2csc(N3CCOCC3)n2)nc1-c1ccc2c(c1)OCO2
	CCPA*	1.50×10^{-0}	-0.1761	OCC1OC(n2cnc3c(NC4CCCC4)nc(Cl)nc32)C(O)C1O
	NECA	6.00×10^{-2}	0.2218	CCNC(=O)C1OC(n2cnc3c(N)ncnc32)C(O)C1O
	ABA-X-BY630*	2.00×10^{-0}	-0.301	F.F.O=C(CCCCNC(=O)COCc1ccc(/C=C/c2ccc(Cc3ccc(-c4cccs4)[nH]3)[nH]2)cc1)NCCCCNc1ncnc2c1ncn2C1OC(CO)C(O)C1O
	CPA	2.30×10^{-2}	1.6383	OCC1OC(n2cnc3c(NC4CCCC4)ncnc32)C(O)C1O
	LUF5834*	9.20×10^{-1}	0.0362	N#CC1=C(N)NC(SCC2N=CC=N2)=C(C#N)C1=C1C=CC(=O)C=C1
	R-IHPIA*	1.30×10^{-0}	-0.1139	CC(Cc1ccc(O)c(I)c1)Nc1ncnc2c1ncn2C1OC(CO)C(O)C1O

	R-PIA	7.80×10^{-2}	1.1079	CC(Cc1ccccc1)Nc1ncnc2c1ncn2C1OC(CO)C(O)C1O
	S-ENBA	5.60×10^{-2}	1.2518	OCC1OC(n2cnc3c(NC4CC5CCC4C5)ncnc32)C(O)C1O
	02-13	5.70×10^{-1}	0.2441	Cc1ccc(-c2nc(-c3ccc(C)cc3)c3[nH]cnc3n2)cc1
	02-19	6.80×10^{-1}	0.1675	Clc1nc(-c2ccccc2)nc2[nH]c(-c3ccccc3)nc12
	02-22	2.90×10^{-1}	0.5376	Clc1nc(-c2ccccc2)nc2[nH]c(C3CCCC3)nc12
	02-32	2.70×10^{-2}	1.5686	c1ccc(-c2nc(-c3ccccc3)c3nc(C4CCCCC4)nc-3[nH]2)cc1
	02-33	3.80×10^{-2}	1.4202	Clc1ccc(-c2[nH]c(-c3ccccc3)nc3nc(C4CCCCC4)nc2-3)cc1
	02-7*	3.00×10^{-0}	-0.4771	COc1ccc(-c2nc(-c3ccc(OC)cc3)c3ncn(C4CCCCO4)c3n2)cc1
	02-35	8.80×10^{-2}	1.0555	Cc1ccc(-c2[nH]c(-c3ccccc3)nc3nc(C4CCCCC4)nc2-3)cc1
Antagonist	[^{3}H]-DPCPX	2.50×10^{-1}	0.6021	[3H]CCC([3H])n1c(=O)c2[nH]c(C3CCCC3)nc2n(C([3H])CC[3H])c1=O
	DPCPX	2.70×10^{-1}	0.5686	CCCn1c(=O)c2[nH]c(C3CCCC3)nc2n(CCC)c1=O
	FSCPX	6.00×10^{-3}	2.2218	CCCn1c(=O)c2[nH]c(C3CCCC3)nc2n(CCCOC(=O)c2ccc(S(=O)(=O)F)cc2)c1=O
	I-BW-A844U	9.30×10^{-2}	1.0315	CCCn1c(=O)c2[nH]c(C3CCCC3)nc2n(CCc2ccc(N)c([125I])c2)c1=O
	LUF5962	2.10×10^{-2}	1.6778	c1ccc(-c2nc(-c3ccccc3)c3nc(C4CCCC4)[nH]c3n2)cc1
	LUF6057*	3.00×10^{-0}	-0.4771	CCCC(=O)Nc1nc(-c2ccccc2)nc(-c2ccc3c(c2)OCO3)c1C#N
	XAC	1.20×10^{-1}	0.9208	CCCn1c(=O)c2[nH]c(-c3ccc(OCC(=O)NCCN)cc3)nc2n(CCC)c1=O

*: Deleted samples with too fast dissociation rates (-log(k_{off}) <0.1)

Table S5. The k_{off} values of 28 p38 MAPK inhibitors

Class/Ref	ID	k_{off} (min^{-1})	-log(k_{off})	Smiles
Thurmond	RWJ67657	3.00×10^{-3}	2.5229	OCCC#Cc1nc(-c2ccc(F)cc2)c(-c2ccncc2)n1CCCc1ccccc1
	RWJ67411	1.20×10^{-2}	1.9208	CCCS(=O)(=O)Cc1nc(-c2ccncc2)c(-c2ccc(F)cc2)n1COCC[Si](C)(C)C
	RWJ67568	6.00×10^{-3}	2.2218	O=C1c2ccccc2C(=O)N1CCCn1c(I)nc(-c2ccncc2)c1-c1ccc(F)cc1
	RWJ67671	1.00×10^{-2}	2	CCCCCCC#Cc1nc(-c2ccc(F)cc2)c(-c2ccncc2)n1CCCc1ccccc1
Regan	10	6.20×10^{-5}	4.2076	Cc1ccc(-n2nc(C(C)(C)C)cc2NC(=O)Nc2ccc(OCCN3CCOCC3)c3ccccc23)cc1C(=O)O
	11	3.80×10^{-5}	4.4202	Cc1ccc(-n2nc(C(C)(C)C)cc2NC(=O)Nc2ccc(OCCN3CCOCC3)c3ccccc23)cc1CN(C)C
	12	2.60×10^{-5}	4.585	CN(C)Cc1cccc(-n2nc(C(C)(C)C)cc2NC(=O)Nc2ccc(OCCN3CCOCC3)c3ccccc23)c1
	13	1.60×10^{-3}	2.7959	Cc1ccc(-n2nc(C(C)(C)C)cc2NC(=O)Nc2ccccc2)cc1
	14	1.20×10^{-4}	3.9208	Cc1ccc(-n2nc(C(C)(C)C)cc2NC(=O)Nc2cccc3ccccc23)cc1
	15	2.30×10^{-5}	4.6383	Cc1ccc(-n2nc(C(C)(C)C)cc2NC(=O)Nc2ccc(CCCN3CCOCC3)c3ccccc23)cc1
	16	3.90×10^{-3}	2.4089	Cc1ccc(-n2nc(C(C)(C)C)cc2NC(=O)Nc2ccc(OCCN3CCOCC3)cc2)cc1
	17	3.40×10^{-4}	3.4685	COc1cc(NC(=O)Nc2cc(C(C)(C)C)nn2-c2ccc(C)cc2)c2ccccc2c1
	18	1.50×10^{-4}	3.8239	COc1cc(NC(=O)Nc2cc(C(C)(C)C)nn2-c2ccc(C)cc2)c2ccccc2c1OCCN1CCOCC1
	5	1.50×10^{-5}	4.8239	CC(C)(C)c1cc(NC(=O)Nc2ccc(OCCN3CCOCC3)c3ccccc23)n(-c2ccccc2)n1
	6	3.80×10^{-4}	3.4202	CC(C)c1cc(NC(=O)Nc2ccc(OCCN3CCOCC3)c3ccccc23)n(-c2ccccc2)n1
	7	4.00×10^{-4}	3.3979	Cc1ccc(-n2nc(C(C)(C)CO)cc2NC(=O)Nc2ccc(OCCN3CCOCC3)c3ccccc23)cc1
	8	3.30×10^{-3}	2.4815	Cn1nc(C(C)(C)C)cc1NC(=O)Nc1ccc(OCCN2CCOCC2)c2ccccc12
	9	2.30×10^{-5}	4.6383	Cc1ccc(-n2nc(C(C)(C)C)cc2NC(=O)Nc2ccc(OCCN3CCOCC3)c3ccccc23)cn1
Millan	1A	4.00×10^{-5}	4.3979	CC(C)c1nnc2ccc(Sc3ccccc3CNC(=O)Nc3cc(C(C)(C)C)nn3-c3ccccc3)cn12
	1AB	2.40×10^{-6}	5.6198	CC(C)(C)c1cc(NC(=O)NCc2ccccc2Sc2ccc3nnc(-c4ccccc4SCCO)n3c2)n(-c2ccc(O)c(Cl)c2)n1
	41	1.40×10^{-2}	1.8539	CCNC(=O)NCc1ccccc1Sc1ccc2nnc(C(C)C)n2c1
Redhead	SB202190	2.70×10^{-2}	1.5686	Oc1ccc(-c2nc(-c3ccc(F)cc3)c(-c3ccncc3)[nH]2)cc1
	SB203580	1.30×10^{-1}	0.8861	CS(=O)c1ccc(-c2nc(-c3ccc(F)cc3)c(-c3ccncc3)[nH]2)cc1
	SB239063	6.70×10^{-2}	1.1739	COc1nccc(-c2c(-c3ccc(F)cc3)ncn2C2CCC(O)CC2)n1
	A2	1.00×10^{-3}	3	Cc1ccc(NC(=O)NC2CC(C(C)(C)C)=NN2c2ccc(C)cc2)cc1
	A3	9.00×10^{-3}	2.0458	Cn1nc(C(C)(C)C)cc1NC(=O)Nc1cccc2ccccc12
	A4	2.80×10^{-2}	1.5528	Cn1nc(C(C)(C)C)cc1NC(=O)Nc1ccc(Cl)cc1
	BIRB796	5.20×10^{-5}	4.284	Cc1ccc(-n2nc(C(C)(C)C)cc2NC(=O)Nc2ccc(OCCN3CCOCC3)c3ccccc23)cc1
